# Dampening and Amplifying Clinical Uncertainty: Middle Actors and Rule-Based Decision Support in Pediatric Oncology

**DOI:** 10.3390/healthcare14213811

**Published:** 2026-11-08

**Authors:** Tamar Yogev, Yael Parag, Michal Singer, Moshe Ashkenazi

**Affiliations:** 1 Adelson School of Entrepreneurship, Reichman University, Herzliya 4610101, Israel; 2 School of Sustainability, Reichman University, Herzliya 4610101, Israel; yparag@runi.ac.il; 3 Edmond and Lily Safra Children’s Hospital, Sheba Medical Center, Ramat Gan 5262000, Israel; singer.michal@gmail.com (M.S.); moshe.ashkenazi@sheba.health.gov.il (M.A.); 4 Dina Recanati School of Medicine, Reichman University, Herzliya 4610101, Israel

**Keywords:** VUCA, uncertainty, Middle-Out Perspective, middle actors, clinical decision support, pediatric oncology, patient safety

## Abstract

**Background:** Volatility, uncertainty, complexity, and ambiguity (VUCA) increasingly characterize healthcare environments, yet most scholarship addresses macro-level leadership rather than the everyday work of frontline professionals. This paper brings an integrated VUCA–Middle-Out lens to clinical informatics through the implementation of AMIT, a rule-based early-alert and workflow-support platform, in a high-volume pediatric hemato-oncology day care unit. **Methods:** In a single in-depth case study drawing on the unit’s own implementation and operational records and a post-implementation nursing survey, we examine how middle actors use rule-based decision support to navigate VUCA conditions in daily practice. Clinical outcomes from a previous evaluation of the same implementation serve as context and are not re-analyzed. **Results:** Re-coding the 61 safety events the platform intercepted in its first 62 working days showed that none arose from a gap in clinical knowledge; all reflected failures in the availability, integrity, transfer, or timing of information the organization already held. Because the platform is designed to surface such failures, this profile characterizes the class of risk it addresses rather than all risks in the unit. Nurses, ward managers, innovation staff, data architects, and the clinical safety team reallocate attention under volatility, aggregate information to reduce uncertainty, restructure information flows to match complexity, and codify digital rules to address ambiguity. Their influence runs mainly downward: middle-down action is documented in all four dimensions, middle-up and sideways actions in two each. Middle actors are not neutral, however—the same enabling, mediating, and aggregating moves that dampen VUCA can amplify it. **Conclusions:** The case suggests five boundary conditions for transferring the Middle-Out Perspective into clinical care, among them a resource condition under which middle actors redirect attention rather than build on the slack the framework prescribes, and yields design principles for platforms that deliberately mobilize middle-actor agency.

## 1. Introduction

Clinical care is increasingly delivered under conditions that management scholars summarize as VUCA: volatility (rapid change whose causes are understood but whose magnitude and duration are not), uncertainty (known causes, but with unclear timing or significance of events), complexity (many interconnected parts and information sources that overload decision makers), and ambiguity (unclear cause–effect relationships and no precedent to guide interpretation) [1,2,3]. Recent work applies this vocabulary to healthcare [4]. Section 2.1 defines the four conditions in detail.

Pediatric hemato-oncology day care makes these conditions concrete in ways specific to the specialty. Chemotherapy doses are calculated from the body surface area, so a mistyped height or weight translates directly into a dosing error, and medication errors in outpatient pediatric chemotherapy are not rare [5]. Repeated invasive procedures, such as lumbar punctures with intrathecal chemotherapy, require current coagulation and blood-count values, while hepatic and renal function determine whether a scheduled cycle may proceed or must be adjusted or deferred. Fever in a neutropenic child is a medical emergency requiring prompt empiric antibiotics [6], and immunocompromised children require strict isolation management. Many clinically decisive results, such as cultures and viral-load assays, are returned days after the visit, when the child is already at home. In a single day care unit these demands arise for many children at once, across several service lines and several unintegrated information systems (Section 3.1). They are the conditions under which the safety events analyzed in this paper occurred (Section 4.2).

Hospitals increasingly respond with clinical decision support (CDS): software that delivers filtered, patient-specific information to clinicians at the point of decision [7,8]. Its measured effects are modest and uneven. Pooling 122 trials covering 1,203,053 patients and 10,790 providers, Kwan et al. [9] found that CDS increased recommended care by only 5.8% (95% CI, 4.0–7.6%) and improved clinical endpoints by a median of 0.3%, although the top quartile achieved absolute improvements of 10% to 62%, significantly larger in pediatric settings and where baseline adherence was low. What distinguishes these more successful interventions remains undefined. Effectiveness depends strongly on how CDS is delivered and integrated into the clinical workflow [7,10].

CDS can also cause harm. Frequent alerts that are mostly irrelevant produce alert fatigue, in which clinicians learn to ignore or override them; in deployed systems up to 95% of alerts have been judged as inconsequential [8]. Reliance on automated checks can erode clinicians’ own scanning and verification skills (deskilling) or lead them to accept system output without scrutiny (over-trust). Rules built for one hospital often fail to work in another, because their information systems, coding, and protocols differ, a problem termed poor transportability. Harm introduced by the information technology itself, rather than by clinical decisions, has been termed e-iatrogenesis [7,8]. Electronic health records (EHRs) compound these risks; interoperability and usability problems persist even in mature national infrastructures [8,11], and poorer EHR usability is associated with higher odds of nurse burnout, job dissatisfaction, and surgical inpatient mortality [12]. Like non-adoption and abandonment, these harms are shaped mainly by implementation, workflow, and the adopting organization rather than by the technology alone [7,13]. Implementation is therefore a meso-level problem: one located at the level of units, departments, teams, and professional groups, between the individual clinician–patient encounter (the micro level) and health-system policy (the macro level).

Oncology illustrates this meso-level problem particularly clearly. Mapping 13 oncology decision-support tools deployed in the European Union and surveying practice across European cancer centers, Frederickx et al. [14] found a fragmented landscape with limited interoperability and inconsistent standards; most institutions still rely on generic EHRs to prepare molecular tumor board discussions, and lack of reimbursement is a critical barrier to adoption. Their proposed remedy, a modular framework, works through cancer centers, professional networks, and health-technology agencies rather than through regulation or individual clinicians, that is, at the meso level. It does not, however, specify which actors carry this work in daily clinical practice.

Much of VUCA scholarship in healthcare and management concentrates on leadership, strategy, and organization-wide responses [3,15,16,17], and says little about how unit-level actors deal with VUCA in everyday work. Clinical informatics does recognize this level through “clinical champions,” “super-users,” “user involvement,” and governance structures, and NASSS (nonadoption, abandonment, and challenges to the scale-up, spread, and sustainability of health and care technologies) asks what work implementation involves, who will do it, and what changes in staff roles, practices, and identities it implies (Table 5 in [13]). These accounts name the relevant actors, but they lack a theory that distinguishes actors by where they sit in the organization—and therefore, by the directions in which, and the means by which, each can exert influence.

The Middle-Out Perspective (MOP) offers such a theory. Developed in energy research and later applied to public health [18,19,20,21], it focuses on middle actors: groups positioned between the “top” (policymakers, regulators, senior management) and the “bottom” (citizens, patients, grassroots initiatives) that have both the will and the means to act. Middle actors can exert influence upward (middle-up), for example by bringing evidence from the ward to hospital management; downward (middle-down), for example by changing how frontline work is organized; and sideways, for example by spreading a practice to peer units. A head nurse who translates a hospital directive into the unit’s daily routine, and reports the resulting problems back to management, acts middle-down and middle-up all at once. Formal middle-out mapping has also revealed overlooked allies in public-health advocacy [22,23]. MOP has not yet been applied to hospital clinical informatics; Section 2.2 sets out the framework in full, and Table 1 defines its key terms.

We apply an integrated VUCA–Middle-Out lens to AMIT (Artificial Medical Intelligence Tool), a web-based, rule-based early-alert and workflow-support platform; despite its name, the version examined here contains no artificial-intelligence or machine-learning component. AMIT was developed in 2022 by the nursing leadership and frontline clinicians of the pediatric hemato-oncology day care unit at Sheba Medical Center, together with the hospital’s innovation center, and was fully implemented in October 2023. It draws data from seven hospital information systems, applies 110 ward-specific rules to identify information that requires action, and displays the results to nurses as alerts, highlighted patient margins, and task lists (Section 3.1). Its clinical evaluation, reported by Singer et al. [24], associated its implementation with fewer urgent pediatric intensive care unit (PICU) referrals, shorter intervals between blood-test availability and treatment, and the interception of 61 safety events in the first 62 working days.

The present paper does not re-evaluate these clinical outcomes. Its contribution is analytical and theoretical: it re-codes the 61 intercepted events by the type of information failure underlying each; maps which professional groups acted on each VUCA condition, in which direction, and how; identifies where the same activity may amplify rather than reduce VUCA; and asks what the case reveals about transferring a framework developed in energy research into clinical care. In doing so it extends an earlier analysis of middle actors addressing VUCA in Israeli and United States energy-innovation ecosystems [25] to the clinical setting. Section 3.2 distinguishes the material reproduced from Singer et al. [24] from the analyses performed for this study.

Because the aim is to elaborate theory rather than to test it, the study is guided by research questions rather than hypotheses [26,27]:

RQ1. How do volatility, uncertainty, complexity, and ambiguity manifest in a pediatric hemato-oncology day care unit, and what type of information failure underlies the safety events that a rule-based platform intercepts?

RQ2. Which middle actors use the platform to address each condition, in which direction (middle-up, middle-down, sideways), and through which mode (enabling, mediating, aggregating); and under what circumstances might the same activities amplify rather than reduce these conditions?

RQ3. What does the case reveal about the conditions under which the Middle-Out Perspective, developed in other sectors, can be transferred to clinical care?

Section 2 sets out the theoretical background of the study, and Table 1 defines the key terms used throughout. Section 3 describes the case, the sources of evidence, and the coding procedure. Section 4 addresses RQ1 and RQ2, Section 5 addresses RQ3 and the amplification part of RQ2 (Section 5.3) and discusses implications and limitations, and Section 6 discusses the conclusions.

## 2. Theoretical Background: VUCA and the Middle-Out Perspective

### 2.1. VUCA as a Multidimensional Lens

VUCA—volatility, uncertainty, complexity, ambiguity—originated in military strategic studies and is widely adopted in management and organizational research [1,2,3]. Bennett and Lemoine [2] distinguished four conditions rather than a generic state of “unpredictable change”: volatility is dynamic change with understood cause–effect relationships and available information but unpredictable magnitude and duration; under uncertainty, cause–effect relationships are known but event significance or likelihood is unclear, which, as Murugan et al. [28] argue for the early COVID-19 response, requires acting on available information—however incomplete—rather than waiting for robust evidence.

Complexity captures environments with many interconnected parts and variables, often producing information overload rather than a fundamental unpredictability [2,15], and ambiguity denotes unclear causal relationships and absent precedents—“unknown unknowns”—that make signals hard to interpret [1].

VUCA thinking has been applied to healthcare and digital transformation with remedies pitched to the physician or the organization as a whole. Cernega et al.’s [4] physician-centered response to the four conditions moves from micro- to macro-management, passing over the meso level; other applications pair each dimension with organization-level strategies such as foresight, co-creation, and continuous learning [16,29]; and leadership-agility reviews center formal organizational leadership [17]. We use Bennett and Lemoine’s [1,2] distinctions to diagnose VUCA within a pediatric oncology day care unit and its response through smart decision support. As in the energy-innovation application [25], the dimensions are analytically distinct but empirically interdependent—a practice that may mitigate one while displacing pressure onto another.

### 2.2. The Middle-Out Perspective

Janda and Parag [18] introduced, and Parag and Janda [19] developed, MOP as a socio-technical framework of “middle actors”: organizations or professional groups sitting structurally and relationally between top actors such as governments, regulators, and senior management and bottom actors such as citizens, patients, and grassroots initiatives; and possessing both agency, the ability to make their own choices, and capacity, the ability to perform them [19]. Position alone is insufficient: many actors between top and bottom neither want to nor are able to act middle-out, lacking the channels, resources, will, or interest to do so [20]. Influence runs upstream toward policy, downstream toward end-users, and sideways across peers and sectors; following Kranzler et al. [20] we call these middle-up, middle-down, and sideways.

Within these directions, Janda and Parag [18] identify three modes of influence, elaborated with public-health examples by Kranzler et al. [20]: enabling opens gates, provides resources, or creates favorable conditions for others to act; mediating covers negotiation, translation, and brokerage between actors, disciplines, or levels, including which evidence is presented upward; and aggregating increases scalability by integrating and institutionalizing ideas or initiatives [20].

MOP has been applied to energy-efficiency building professionals, middle actors in energy-system transitions, an Israeli organization linking government, firms, investors, municipalities, and communities in a renewable-energy ecosystem, and intersectoral public health programs coordinating resources across agencies [18,19,20,30], while UK cases show that formal middle-out mapping broadens the range of recognized allies [22,23].

Three qualifications are essential. First, the middle is dynamic and relational: an actor may be top, middle, or bottom depending on the process, as a local leader may regulate locally but advocate nationally [20]. Second, middle actors are not neutral: they may facilitate beneficial change but can also be mobilized to obstruct it [22,23], and their interventions may themselves amplify VUCA conditions [25]. Third, middle-out action complements top-down authority and bottom-up mobilization, and structural and political constraints can limit its effectiveness [20].

### 2.3. Integrating VUCA and the Middle-Out Perspective in Clinical Informatics

In a study on energy entrepreneurship, Parag and Yogev [25] integrated VUCA and MOP to analyze meso-level organizations in innovation ecosystems: the middle actors examined used aggregating, mediating, and enabling strategies to help others navigate VUCA conditions, while their interventions could also generate fragmentation and hype-driven cycles. Proposing MOP to be widely applicable to other innovation domains facing VUCA conditions, health-tech among them, the study invites the present extension; we retain its three action-mode definitions unaltered so that the two analyses remain comparable.

Applying this lens to clinical informatics addresses a theoretical gap. Kawamoto et al. [10] identified automatic provision within clinician workflow, recommendations rather than assessments, delivery at the time and place of decision making, and computer-based delivery as independent predictors of improved clinical practice; Berner [7] summarizes effective CDS delivery through the five rights of information, person, format, channel, and time; and NASSS explains the implementation difficulty due to complexity across seven domains, from the condition and the technology to the adopter system, the organization, and mutual adaptation over time, finding that programs complex in several domains rarely became mainstream [13]. Yet none of these accounts, nor Frederickx et al.’s [14] emphasis on stakeholder collaboration, conceptualizes the positional and directional influence available to ward nurses, innovation units, or quality departments. MOP supplies the positional vocabulary, while VUCA specifies the conditions toward which influence is directed.

Pediatric oncology day care units are VUCA-prone micro-ecosystems: volatility arises from fluctuating child health and workloads; uncertainty from delayed or unseen results and evolving protocols; complexity from fragmented information technology (IT) and multi-actor workflows; ambiguity from deterioration patterns and evolving digital roles. Nurses, ward managers and clinical leads, innovation staff, IT and data architects, and the clinical safety team may function as middle actors between senior management and individual patients, and their position is relational [20]: a nursing team may be bottom relative to national digital-health regulation, middle relative to platform governance, and top relative to an individual patient encounter.

## 3. Methods

### 3.1. Case Study Design and Case Selection

This is a single, in-depth case study [27] of AMIT’s implementation in the pediatric hemato-oncology day care unit at the Edmond and Lily Safra Children’s Hospital, Sheba Medical Center. It is in-depth in two senses: it draws on several independent sources of evidence (Section 3.2), and it has unusually complete access to rule provenance and implementation decisions. The case was selected purposively, for theoretical leverage rather than representativeness [27]: it is unusually well documented, and it is a compressed, observable meso-level case of VUCA and middle-out dynamics. The unit is a high-volume tertiary service treating approximately 120 patients daily with fifteen nurses per shift. AMIT was developed in 2022 by the unit’s nursing leadership and frontline clinicians with ARC (Accelerate, Redesign, Collaborate), Sheba’s innovation center; staff were trained between January and September 2023 and the platform was fully implemented from October 2023. The four phases by quarter (Q)—pre-intervention Q4 2021–Q1 2022, immediate post-implementation Q4 2023–Q1 2024, and two follow-ups through Q3 2025—cover 55,986 visits: 12,143, 10,087, 10,278, and 23,478. The name AMIT stands for Artificial Medical Intelligence Tool; despite the name, the version examined here contained no artificial-intelligence or machine-learning component and operated solely on predefined rules. Capabilities based on large language models are in development and were not in use at any point in the study period (Section 4.7).

Implementation proceeded in stages. Nurses were trained first, on an interface designed to be intuitive so as to lower the training burden. Rules were tested during a trial period before activation, and thresholds were calibrated when alerts proved too sensitive; for example, the threshold for an alert that staff perceived as generating false positives was adjusted slightly. After implementation, most nurses used the platform routinely, and three physicians joined as regular users after the initial implementation period. In the taxonomy of the CDS literature, AMIT is a knowledge-based, locally developed (“homegrown”) system delivering primarily non-interruptive, screen-based alerts and worklists at the point of care [8].

AMIT runs on 110 rules. They were specified by the unit’s chief nurse and the nursing team, and each rule was approved by the ward director and the head nurse before activation. Most clinical rules were transcribed from the unit’s existing, validated clinical protocols; many others support administrative nursing work, for example by surfacing the type and severity of a documented allergy, which is difficult to establish from the electronic record. Table 2 shows the representative clinical rules. All rules follow a single operating principle—that a rule should fire only when the information it surfaces must be acted on—intended to minimize false-positive alerts, and their thresholds are bounded institutionally within the panic values that Sheba Medical Center defines for laboratory, imaging, and pathology results. Quality assurance by the innovation center consisted of sample checks on laboratory, imaging, and pathology results to confirm that AMIT had alerted about them, as a guard against false negatives. After implementation, only minor changes were made, mainly to thresholds. No scheduled review of the rule base was established beyond the approval of each new rule. Alert firing rates, override rates, and the number of active rules at the end of follow-up were not recorded, and no system logs exist from which they could be reconstructed, so no measure of realized alert precision is available.

### 3.2. Sources of Evidence

The analysis draws on five bodies of material generated by the unit while developing, implementing, and operating the platform (Table 3). All available material of each type for the periods shown was considered, and none was sampled. Two of the authors led the development and implementation of AMIT and its clinical evaluation [24], which gave the team direct access to these records; the other two authors had no role in developing, implementing, or evaluating the platform and carried out the coding (Section 3.5).

Four of the five are unit records—platform and rule documentation, confirmed incident records, timestamped electronic health-record operational data, and implementation and training documentation—each described in Supplementary Material S1. The fifth is a post-implementation structured nursing survey: ten Likert-type items in Hebrew on a fully labeled five-point scale (1 = strongly disagree, 5 = strongly agree), administered anonymously online through SurveyMonkey to the twenty-two nurses in the unit. The head nurse distributed the link by WhatsApp on 1 July 2025, about 21 months after full implementation, with a written invitation explaining the purpose of the survey, the scale, its anonymity, and that participation was voluntary; the scale was also explained orally. The survey remained open for three weeks; nineteen nurses completed it, yielding a response rate of 86.4%. The instrument is reproduced in the English translation as Supplementary Material S2. The incident records and operational data are those whose clinical results are reported in Singer et al. [24]; the present paper re-codes and re-interprets them. Table 3 distinguishes what was reproduced from that evaluation from what was analyzed for this study; clinical outcome statistics in Section 4 were taken from Singer et al. [24] as context and were not re-analyzed.

### 3.3. Ethics Approval and Access

The AMIT implementation and its evaluation were approved by the institutional review board of Sheba Medical Center (approval no. 1208-24-SMC, dated 11 September 2024), and the approval covers the secondary use of the operational and safety records analyzed here. The committee granted a waiver of informed consent for this secondary use. The approval is renewed annually and covers the full follow-up period. The paper analyzes those records at the organizational level, with no additional patient data and no new patient contact, and incident descriptions were de-identified before analysis. The use of the records was authorized by the ward director as part of the approval. The nursing survey was an anonymous staff-attitude survey, which under Sheba Medical Center policy does not require ethics committee approval; participation was voluntary and anonymous, as stated in the written invitation (Supplementary Material S2), and no identifying information was collected.

### 3.4. Analytical Framework

Our framework combines Bennett and Lemoine’s differentiated VUCA lens [1,2] with MOP [18,19,20]. Volatility, uncertainty, complexity, and ambiguity are operationalized as distinct conditions, classified by Bennett and Lemoine’s definitions; middle actors’ directions as middle-up, middle-down, and sideways; and their modes, following Parag and Yogev [25], as aggregating, mediating, and enabling.

**Figure 1:**
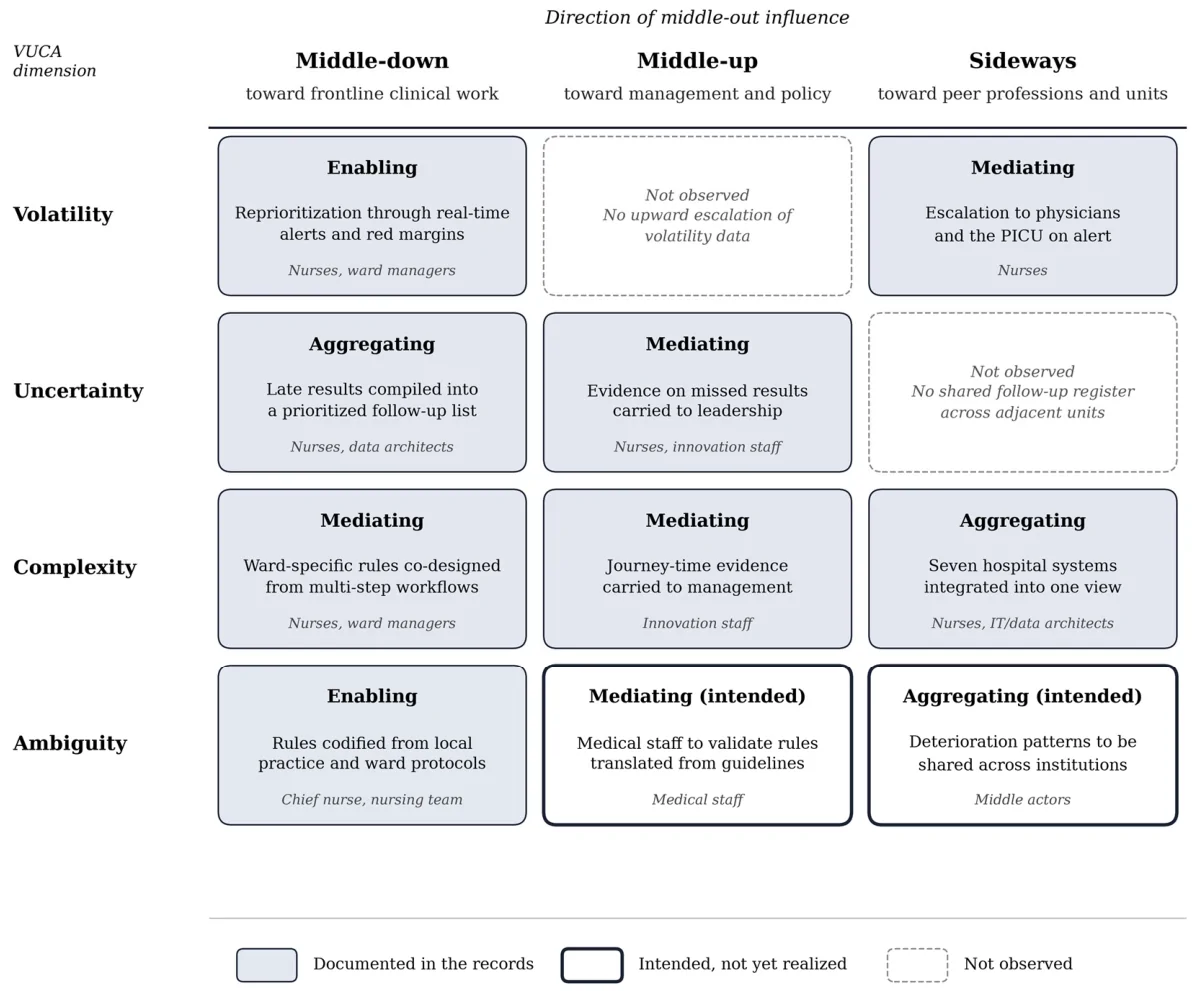
An analytical matrix of VUCA dimensions, directions of middle-out influence, and modes of action in the AMIT case. Each cell gives the dominant mode of influence, the activity, and the middle actors involved, and is shaded by evidential status. Outcome statistics from Singer et al. [24] are reported in the text rather than in the figure. The figure was prepared with the assistance of Perplexity.

Building on the energy-sector study, our matrix crosses VUCA dimensions with middle-out directions and modes (Figure 1). For each coded excerpt we ask which VUCA dimension it exemplifies; which middle actors address it; in which direction—middle-up, toward management and institutional policy; middle-down, toward individual decisions and episodes of care; sideways, toward professions and units—and in which mode they act; and whether poorly calibrated activity could amplify rather than reduce VUCA.

Direction denotes the level at which influence is aimed, not the type of person: acting on a bedside alert is middle-down, and one actor can act in all three directions within a shift (Section 2.2). Middle-down points to frontline clinical work, not to patients, who benefit but do not participate. AMIT is treated throughout as an instrument that middle actors use, not as a middle actor itself, and because most records derive from AMIT, off-platform work such as corridor conversation and handover is under-registered.

Position alone does not make a group a middle actor [20]. We therefore treated a group as a middle actor in a given process only if it met three criteria: (i) it sits between the actors who set the terms of care, such as hospital management and regulators, and those who receive it, namely patients and families; (ii) it controls a resource that others need for that process, such as clinical information, a technical system, a workflow, or the formal recognition of an event; and (iii) the records document that it used, or sought to use, that resource to influence other actors. Groups meeting the first criterion but not the third were treated as positional middle actors that did not act as such in the process examined.

### 3.5. Coding Procedure

Coding was carried out by the two authors who had no role in developing or implementing AMIT, following the procedure of their earlier energy-sector study [25] in three theory-guided steps with large language models (ChatGPT and Perplexity, paid versions) as assistants: extraction of excerpts from the implementation records; assignment of those excerpts to the four VUCA dimensions using Bennett and Lemoine’s [1,2] definitions; and coding of the VUCA-tagged excerpts for actors, influence directions, and modes [18,19,20]. The models proposed excerpts and assignments; the authors accepted, corrected, or rejected each proposal based on the original material, and every final coding decision is the authors’. The two authors who developed AMIT then reviewed and approved the coded assignments and compared the findings against the original records. The coded matrix underpins Figure 1 and the thematic narrative of Section 4; operational data (visits, referrals, and journey times) were not coded and are reported as context. Supplementary Material S3 provides the codebook, with a decision rule and an example for each category.

Contested or ambiguous assignments were settled against the original records rather than by the models, and Figure 1 reports the dominant mode in each cell. Each cell in Figure 1 is also classified by its evidential status: documented in the records, intended but not yet realized, or not observed. Planned capabilities, such as those based on large language models and the intended sideways diffusion of the platform, are identified as such in the text and in Section 5.3, and statements resting on the platform team’s account rather than on the records are attributed to that account.

A further pass, carried out by the same two authors, re-coded the 61 intercepted safety events by the type of information failure underlying each, rather than by clinical domain, producing the four categories reported in Section 4.2. Each event received a single category, according to the decision rules given in Section 4.2. Four groups of assignments, covering fourteen events, were contested; they were adjudicated by the co-author who established and led the platform, working with the underlying event records, and the reasoning for each group is set out in Supplementary Material S3. One event fit none of the four categories initially defined, and the fourth category was widened to accommodate it. The two potentially life-threatening events had been classified as such by a senior physician in the original evaluation [24].

Only de-identified material was processed with the language models: incident descriptions had been de-identified before they were made available for analysis, and no patient identifiers were entered into either tool.

Two authors developed and implemented AMIT. This gave the analysis unusually detailed access to rule provenance and implementation decisions, but it also created a risk of interpretive bias. We addressed this risk first by separating the coding role from the implementation role, as described in the first paragraph of this section. In addition, coding followed an explicit codebook with a decision rule for each category (Supplementary Material S3); every assignment was checked against the original records, and each cell of Figure 1 was classified by evidential status. Because no coder outside the author team was used, no inter-rater agreement statistic is available (Section 5.6).

## 4. Results

### 4.1. The Middle Actors in the AMIT Case

Because the analysis depends on who occupies the middle, we identify the actors and their positional bases. Five groups recur; each is middle not by seniority but because it sits between those setting the terms of care and those delivering it, holding something both need.

Oncology nurses are the operative middle. Fifteen per shift coordinate all unit services, hold the only continuous patient view across a treatment day, and were the platform’s intended primary users—placing alert interpretation with the group that has the least formal protocol-changing authority and the most continuous contact with consequences.

Ward managers and clinical leads approve routine changes and bring cases requiring mandates or resources to management. ARC innovation staff sit between the clinical team and the technical estate, with a remit to build but no prescribing authority. IT and data architects control the seven source systems and thus visibility. The clinical safety team confirms reported incidents, converting locally surfaced events into institutionally recognized safety events. These positions differ in what they control rather than in rank: each holds a different link from source data to action and its recognition. Authority over the rule base sits inside this middle rather than above it, bounded only by the hospital’s panic values (Section 3.1), and no refusal of a candidate rule is recorded.

Applying the criteria in Section 3.4 identifies these five groups and excludes others. Patients and families are bottom actors: they are affected by middle-actor decisions but do not take part in them. Senior hospital management is a top actor, the recipient of middle-up evidence rather than a participant in platform governance. Physicians illustrate the third criterion: they occupy a middle position. But their reluctance to adopt an additional screen led the platform to be placed at the nursing station (Section 4.8), and only three physicians joined as regular users after the initial implementation period; with respect to this instrument they acted mainly as recipients of aggregated results rather than as middle actors shaping it. More broadly, the multi-month implementation interval described in Section 3.1 amounted to an investment in middle-actor capacity rather than a purely technical roll-out.

### 4.2. The Informational Profile of the Intercepted Events

We first examine the profile of events the platform intercepted, because it characterizes the informational conditions on which all four responses operate. In the first 62 working days after full implementation, approximately the first three months, the platform surfaced 61 safety events that the clinical safety team confirmed; these are all the confirmed events of that period. The unit records them by clinical domain—height and weight errors, electrolyte and metabolic abnormalities, invasive procedures proceeding despite abnormal coagulation or bilirubin values, isolation management during a norovirus outbreak, and an overlooked cytomegalovirus result—the form in which safety data are ordinarily reported and audited.

All 61 were re-coded, by the procedure in Section 3.5, not by clinical domain but by the information failure underlying each. Table 4 reports the result.

The distribution reveals a predominantly informational rather than clinical-knowledge problem. None of the 61 events intercepted by AMIT reflected a clinical-knowledge gap; the required knowledge was available, but the movement, integrity, or timing of information failed, and the first two categories together account for 57 of the 61 events. Because AMIT was designed to move and check information the organization already holds, it surfaces information failures by construction and could not detect a clinician acting on incorrect knowledge; and events the platform did not surface are absent from the data. The profile therefore characterizes the class of risk the platform addresses, not all risks in the unit. The pattern, consistent with estimates attributing large annual sums in the United States health system to failures of care delivery and of care coordination rather than to deficits in clinical knowledge [31], points to organizational interfaces and information flows as the sites of intervention.

### 4.3. VUCA Manifestations and Middle-Out Responses in the AMIT Case

The interfaces identified in Section 4.2—between hospital systems, units, and the point of care—are the positions the five middle-actor groups occupy. The question is therefore how the four VUCA conditions appear in this unit, and which groups acted on each, in which direction and mode. Figure 1 identifies the dominant mode in each cell and whether the activity is documented, intended, or not observed.

### 4.4. Volatility: Early Alerts and Agile Prioritization

In AMIT, volatility is the rapid, sometimes unpredictable clinical change—particularly in neutropenic children at risk of life-threatening infections—together with fluctuating caseloads and acuity. Without dedicated support, nurses continuously scan multiple systems and rely on memory and ad hoc coordination to identify deteriorating patients.

AMIT enables nurses’ and ward managers’ middle-down action: real-time vital-sign and lab alerts and red margins for high-risk patients support reprioritization. The unit holds none of the spare capacity or stockpiled resources that Bennett and Lemoine [2] prescribe for volatility and that NASSS treats as an indicator of the capacity to innovate [13] (domain 5A). It innovated in their absence, reallocating attention across fixed staff and hours toward patients likely to deteriorate. Sideways mediation among nurses and physicians accompanied earlier intervention and, as reported by Singer et al. [24], a 78% relative reduction in urgent PICU referrals—among children treated with broad-spectrum antibiotics, 13 of 52 (25.0%) were urgently referred to the PICU before the intervention compared with 4 of 72 (5.6%) in the final follow-up (*p* = 0.01 across the four phases; *p* = 0.004 for the probability of deterioration in a logistic regression). Antibiotic administration within three hours rose from 51.9% to 69.4% (27 of 52 to 50 of 72), a relative improvement of 33.7% or 17.5 percentage points, which did not reach conventional significance (*p* = 0.167 across the four phases). These before–after comparisons come from an uncontrolled evaluation and do not establish causation. Singer et al. [24] report that patient characteristics (sex, age, proportion of foreign patients, prior-year visits, and high-risk diagnoses) did not differ significantly across the four phases (all *p* > 0.3), and that seasonal variation in patient mix was ruled out; according to the platform team, staffing and clinical protocols did not change during the study period, the pre-intervention period was chosen as it was unaffected by the COVID-19 disruption, and war-related disruption of activity was limited to the first week of October 2023. Residual confounding by unmeasured factors cannot be excluded. The middle-out reading concerns response, not magnitude.

One direction is absent. We found no evidence that bedside volatility data—alert frequency, distribution of high-risk margins, timing of deterioration—were aggregated and carried upward as a picture of unit operating conditions. The platform reorganized volatility handling within the ward, but not how volatility was represented above it.

### 4.5. Uncertainty: Aggregating and Surfacing Delayed Results

Uncertainty arises when clinically significant tests—blood and urine cultures, cytomegalovirus and other viral load assays, tuberculosis screening, or computed tomography reports—return days or weeks after the prompting visit and it is unclear whether anyone knows. Their clinical meaning is understood, but their timing and visibility are not, mirroring Bennett and Lemoine’s [2] definition of uncertainty.

Middle actors use AMIT to gather, interpret, and share information. ARC innovation staff and IT/data architects extract and aggregate late-arriving results from multiple systems; physicians and nurses use prioritized alerts for follow-ups. Aggregation meant that positive cultures, abnormal imaging, and significant serological changes reached physicians whether or not the patient was in the clinic—a function physicians valued because it lies outside routine encounter-based care—and the same staff carry evidence of missed and delayed results upward, where it supports the case for mandate and resources. Otherwise continuity erodes, because the ordering clinician is often not on duty when a result arrives. These middle-down aggregation and middle-up mediation moves address a structural rather than individual failure: AMIT converts a task implicitly delegated to individual memory into an aggregated, institutionally owned process.

This aggregation stops at the unit boundary. We found no shared follow-up register spanning the day care unit and the adjacent wards, clinics, and services with which it exchanges patients, so a result arriving after a patient moves on remains the responsibility of the unit holding the record. The two coordination and hand-off failures in Table 4 are the smallest-scale instance of the same problem.

### 4.6. Complexity: Integrating Fragmented Systems and Workflows

The pediatric oncology day care unit is structurally complex. It operates outpatient clinic, long-term follow-up, chemotherapy, transfusions, sedation, stem cell collection, and emergency room services through seven unintegrated IT systems that staff navigate under intense time pressure: Chameleon, Mega Lab, Q-Flow, blood bank, pathology, imaging, and Cato chemotherapy ordering and delivery software. The event profile in Section 4.2 shows these failures to be ones of visibility, integrity, transfer, or timeliness across these systems, tractable to meso-level integration rather than clinical education. Its everyday cost is decision fatigue: reconciling interfaces under time pressure risks overlooking laboratory values, medication discrepancies, or isolation requirements. This is VUCA complexity [2,15].

Middle actors—nurses, ward managers, IT/data architects, and ARC innovation staff—act in all three directions here, the only dimension in which they do so: sideways aggregation, middle-down mediation, and middle-up mediation. AMIT’s dashboard integrates seven systems and filters data through ward-specific rules that nurses and managers co-design, translating complex multi-step workflows into manageable alerts and tasks. The mean interval between blood-test availability and the administration of electrolytes or transfusions fell, as reported by Singer et al. [24], from 104 ± 45 to 44 ± 32 min in the first post-implementation period, a reduction of 58% (*p* < 0.001). Innovation leaders carried this evidence to hospital management in support of sustaining and extending the platform. Integration here precedes harmonized standards; locally negotiated rules and point-to-point interfaces deliver visibility within the unit while reproducing fragmentation through bespoke logic, and specialist capability exists only weakly in available innovation and data staff.

### 4.7. Ambiguity: Codifying Digital Rules and Anticipating Deterioration

Ambiguity in AMIT is evident in deciding which digital rules detect deterioration early and how digital alerts should be embedded in responsibility structures: it is not always clear which combinations of vital signs, lab values, and contextual factors best indicate impending decline. These situations resemble Bennett and Lemoine’s [2] ambiguity, a lack of knowledge about the basic “rules of the game.”

Middle actors respond by codifying rules rather than through a cycle of trial and revision. Rules are pre-defined from local practice and aligned with ward-specific protocols and standard operating procedures, and one is documented in detail: a red margin is applied to every neutropenic patient with at least one abnormal vital sign, which Singer et al. [24] describe as a beacon that illuminated those patients. This middle-down enabling puts rules with those who act on them, and authority over the rule base is locally held but institutionally bounded (Section 3.1 and Section 4.1). A planned capability based on large language models, in development and not in use during the study period, would translate guidelines and standard operating procedures into executable rules for validation by medical staff, placing the approval of a codified rule outside the nursing middle that operates it. Both the right rule and its weight remain unsettled: under-reliance leaves AMIT ignored; over-reliance substitutes it for independent clinical judgment. Middle actors intend to use AMIT to aggregate patient-specific deterioration patterns, enabling sideways learning across institutions and narrowing specific rule uncertainties over time.

This ambiguity is not transitional; it is a standing condition requiring a permanent middle-actor function, which makes ownership of rule revision a governance question rather than a technical one.

### 4.8. Staff Experience, Placement, and Mandate

This subsection cuts across the four dimensions, taking staff experience as a vantage point for all of them. A post-implementation nursing survey of the unit’s twenty-two nurses was completed by nineteen (86.4%). Items were answered on a fully labeled five-point scale (1 = strongly disagree, 5 = strongly agree), and responses indicating four or five are treated as agreement; Table 5 reports the full distribution for all ten items. All nineteen rated their frequency of use at four or five. Agreement was unanimous that AMIT reduced the likelihood of missing critical medical data, increased confidence in patient-management decisions, and would be recommended to colleagues (19 of 19 each); all 18 nurses who answered the item agreed that it improved their ability to prioritize clinical tasks. Seventeen of nineteen (89%) agreed that it influenced their clinical day, improved workflow, enhanced clinical communication, decreased end-of-shift bottlenecks, and enabled faster patient transition; the other two chose the midpoint. No respondent chose one or two on any item. These are staff perceptions, collected about 21 months after implementation and without a pre-implementation baseline, not objective measures of improvement.

From the Middle-Out Perspective, nurses’ reported prioritization and reduced likelihood of missing data suggest that AMIT strengthens agency and capacity in volatile, uncertain, and complex environments [19], while fewer bottlenecks and better task ordering reflect middle-down enabling and mediating action.

The most theoretically consequential detail concerns the reason why the platform sits at the nursing station. Singer et al. [24] explain that the placement was due to the physicians’ reluctance to use an additional screen; and the platform team’s account adds a premise about sequence—nurses are the middle of the middle in this unit, and adoption by nurses was expected to carry physicians with it, whereas the reverse sequence was not considered feasible. Either way, the platform was optimized for nurses at their workstations rather than embedded into physician workflow, and reluctance at an adjacent professional level is the clinical instance of Kranzler et al.’s [20] dynamic middle, where not every positional middle actor has the will or resources to act. But it concentrates surveillance work, and with it de facto accountability, at the nursing station while the records document no change in nursing responsibilities, workload, or mandate—a shift in practice unmatched by a documented shift in mandate.

Nurses’ positioning as primary users and informal early adopters suggests they may diffuse digital innovations sideways across professional networks, a role highlighted in middle-out analyses of energy transitions and public health programs [18,20,23]. Staff experience is thus a vantage point on how middle actors appropriate and sustain such platforms, and not merely an outcome variable. This sideways diffusion is an intention, not a documented spread.

## 5. Discussion

### 5.1. What the Platform Managed

AMIT, as configured and used by middle actors, addresses each condition distinctly: attention is reallocated under volatility, information aggregated to reduce uncertainty, information flows restructured to match complexity, and rules codified to bound ambiguity. Two follow Bennett and Lemoine’s [2] prescriptions directly—gathering information beyond existing sources for uncertainty, and matching internal operations to external complexity—while two depart from them, since they prescribe building slack for volatility and experimentation for ambiguity, whereas this unit reallocated attention within a fixed establishment (Section 5.2) and codified rules in advance. Smart rule-based platforms can thus be designed as explicit VUCA-management instruments in clinical care. Against CDS evidence this is a mechanism, not a magnitude, claim. Why a minority of interventions achieve substantial rather than marginal improvement remains undefined [9], and the VUCA–Middle-Out lens offers one explanation—platforms must match their VUCA condition, and middle actors must retain the agency and capacity to keep them matched.

The re-coding in Section 4.2 specifies what these middle actors were managing: not gaps in clinical knowledge but failures in the availability, integrity, transfer, or timing of information the organization already held (Table 4). Because the platform was built to detect exactly such failures, this profile is evidence on the class of risk AMIT addresses rather than a census of risks in the unit. This locates the unit’s uncertainty in its information infrastructure rather than in clinical competence, and explains why an instrument that moves and checks information the organization already holds was the effective response. It also locates middle-actor work at the interfaces between systems, units, and shifts, where information stalls because no single actor holds both the data and the mandate to act.

### 5.2. Boundary Conditions on Transferring the Middle-Out Perspective

Setting this case beside the analysis of middle actors in energy entrepreneurship reported by Parag and Yogev [25] is instructive less for shared structure than for divergence. In both, middle actors enable, mediate, and aggregate to clarify signals, orchestrate networks, and stabilize practices; here oncology nurses, ward managers and clinical leads, innovation staff, IT and data architects, and the clinical safety team do so through alerts and rules, coordination across units and systems, and aggregation of safety-event and performance data.

The divergences do more work and are the substantive finding: the case suggests that the Middle-Out Perspective transfers into clinical care only under five boundary conditions absent in energy. First, temporal: energy intermediaries work across policy cycles and funding rounds over months to years, whereas pediatric oncology middle actors work at shift scale, within minutes to hours, so their strategies are real-time triage and micro-coordination rather than medium-term ecosystem shaping. Second, risk and its bearer: energy failure means startup collapse, misallocated capital, and fragmented ecosystems; here it means missed deterioration, delayed antibiotics, procedural error, and patient harm. Energy’s bottom actors—startups and entrepreneurs—participate and can respond, whereas patients and families are affected by middle-actor decisions without participating in the arrangements producing them, so middle-out analysis in healthcare carries an ethical dimension it does not carry in energy—one that also weighs on the actors themselves, since the coupling that can lighten their burden can equally intensify it. Third, the medium of influence: energy intermediaries convene through forums, roadmaps, and ecosystem maps, with instruments separable from practice, whereas embedded point-of-care software couples alerts immediately to clinical action, raising the returns to correct rules and the costs of wrong ones—alert fatigue, rule proliferation, non-traveling local logic, and over-trust eroding independent judgment. Fourth, and most consequential for theory, the source of mandate: the independent energy intermediaries construct their own, drawing trust from their autonomy from formal state structures and exercising influence through convening rather than delegated accountability, while even the federally embedded intermediary derives legitimacy from funding and national scope rather than from clinical accountability [25], whereas clinical actors work in a licensed, accountability-bearing hierarchy where authority is delegated and each automated rule owned by a named professional. What energy treats as reflective strategy becomes formal governance here: durable mandates for rule revision and maintenance, and a specified locus of human oversight, without which a framework built on self-constituted intermediaries does not describe this situation.

The fifth condition is resource endowment. Parag and Yogev [25] drew this contrast within the energy sector itself: the US intermediary worked inside a resource-rich, federally anchored innovation system, while the Israeli actors worked in a more resource-constrained but socially dense one, achieving comparable stabilizing effects through rapid convening, relationship capital, and flexible coordination. Both could nonetheless enlarge their field’s resources by drawing in new participants, programs, and funds. A pediatric oncology day care unit running at capacity cannot add staff, hours, or reserve capacity. Middle actors instead reallocated attention across a fixed establishment, directing the same staff and hours by rule toward the patients most likely to deteriorate. On the platform team’s account, the time this required was released by disrupting the traditional patient journey, reorganizing sequential steps to run in parallel rather than by adding resources. The same constraint shapes rule handling: writing a rule is a single act, whereas revision is recurring work falling to the establishment already delivering care, so the iterative element remains a plan rather than a practice in use. Where middle actors cannot expand what an organization has, their influence operates by redirecting it, and a rule-based platform is the instrument of that redirection.

Two conditions leave visible traces in the case. Middle-down activity is documented in all four dimensions, whereas middle-up and sideways activity is each documented in two—the distribution a delegated mandate would produce. Figure 1’s two cells with no observed activity fall where they predict: no upward escalation of volatility data, and no sideways coordination in the uncertainty row. Moreover, both documented middle-up actions carried evidence to management for a mandate or resources rather than exercising independent authority. Delegated mandate does not mean the absence of local authority: the unit wrote its own rule base and its ward director approved it, but within thresholds fixed by the hospital’s panic values and without a durable mandate for the recurring work of revision (Section 4.7). Actors petition upward for mandate and resources; they do not redefine upward. Absence of documented practice in unit-generated materials is not non-occurrence, but the gaps are consistent with the temporal and mandate conditions and identify positions to examine first in further clinical application.

Each condition combines a documented feature of this case with a theoretical interpretation. The compressed timescale, the instrument’s coupling to clinical action, the delegated mandate, and the fixed establishment are documented in the unit’s records (Section 3.1, Section 4.1, Section 4.4, Section 4.5, Section 4.6 and Section 4.7); that they operate as boundary conditions on the framework is our interpretation, derived from contrast with a single energy-sector study [25]. We expect the conditions to apply most directly in other high-acuity, protocol-driven settings where frontline staff act on shared digital information under time pressure, such as intensive care units, emergency departments, and adult oncology day units, and less directly where clinical tempo is slower, as in chronic-disease management, where middle actors may work closer to the energy-sector pattern. These are propositions for comparative research (Section 5.6), not established generalizations.

### 5.3. When Middle Actors Amplify VUCA

Middle-out strategies can reduce VUCA yet create fragmentation, hype, or crowding [25]. AMIT’s implementation was associated with improved safety and efficiency through filtering and prioritizing data, but rule and alert dependence can under-flag signals or generate noise. Its design principle was that a rule should fire only when the information it surfaces must be acted on (Section 3.1). Because alert firing and override data were not recorded, and no logs exist from which they could be reconstructed, the precision actually achieved cannot be verified, and alarm fatigue remains an ongoing challenge: up to 95% of deployed-system alerts were judged inconsequential, and physicians often distrust or do not read them [8]. In 112 ambulatory clinicians, reminder acceptance dropped by 30% for each additional reminder per encounter and 10% for each five-percentage-point increase in within-patient repeats, while the sheer amount of work had no independent effect [32]. The risk is alert-stream density and redundancy, a middle-actor design variable, rather than patient volume. Nurse practitioners were four times as likely as physicians to accept drug alerts [32], which is consistent with, though not evidence for, AMIT’s placement at the nursing station (Section 4.8). Berner [7] notes that frequent inaccurate alerts, often from stale records rather than faulty logic, lead clinicians to discount all CDS advice, and that most health-IT harms—“e-iatrogenesis”—come from implementation and workflow failures, not erroneous recommendations. Middle actors therefore need continuing roles in rule design, validation, revision, and oversight. Table 6 sets out the activities in the AMIT case that could amplify rather than reduce VUCA.

As AMIT moves toward integrating large language models to translate guidelines into rules and learn patient-specific deterioration patterns, it raises questions of accountability, transparency, and equity; middle actors must mediate algorithmic outputs, clinical judgment, and policy so as to enhance rather than undermine agency and trust. Frederickx et al. [14] report that Watson for Oncology concordance with human decisions did not ensure use—absent retrospective learning, thin training data for some cancers, missing patient-specific and ethnic variation, and US-specific approvals limited transferability rather than accuracy, and competing oncology tools produced different recommendations from identical data through discrepant interpretive frameworks. Governing this variability is a middle-actor mediating task [19,20].

### 5.4. Theoretical Contributions

The integrated VUCA–Middle-Out lens makes four theoretical contributions. First, it extends MOP to hospital-based clinical informatics, showing VUCA and middle-actor dynamics embedded in high-acuity micro-workflows and identifying oncology nurses and innovation teams as platform-shaped middle actors. Second, it refines VUCA scholarship by showing the distinct conditions managed in everyday clinical work, where real-time, safety-critical contexts compress middle-out strategies and intensify moral pressure, cognitive load, and questions of professional autonomy; and by showing that middle-actor practice is two-directional: the same moves that dampen these conditions can amplify them (Section 5.3). Third, it proposes five boundary conditions under which a framework developed in energy research transfers into clinical care (Section 5.2). Fourth, it supplies clinical informatics with what its own frameworks lack; Kawamoto et al. [10] and Berner [7] specify how support should be delivered and Greenhalgh et al. [13] why programs fail to scale, whereas MOP specifies who can act and in which direction, turning implementation guidance into an account of distributed agency.

### 5.5. Design Principles for Middle-Actor Platforms

Our analysis, read together with the CDS literature, suggests five design principles for smart, rule-based platforms in high-acuity care. First, tailor platforms to the needs, constraints, and capacities of middle actors—especially nurses and innovation teams—as primary users and co-designers, doing CDS with users rather than to them [7]. Second, match rule logic to VUCA: attentional reallocation for volatility, information for uncertainty, restructuring for complexity, and codification with scheduled revision for ambiguity. Deliver these as interruptive alerts, non-interruptive worklists, aggregated dashboards, and explicitly provisional monitored rules respectively, rather than as one undifferentiated alert stream [7]. Third, sustain the capacity to enable, mediate, and aggregate over time using named rule-base ownership, scheduled review with alert-precision and override metrics, a retirement path for noisy rules, and fewer within-patient repeat alerts [8,32]. Fourth, perform middle-out mapping formally at the project outset, because it surfaces non-traditional allies—procurement, biomedical engineering, infection control, patient representatives—otherwise invisible to the implementation team [22,23]. Fifth, where a platform is directed at one professional group, formally sanction the resulting transfer of surveillance work and accountability; otherwise a de facto middle actor operates without mandate, creating equity and durability risks.

### 5.6. Limitations and Future Research

This study has several limitations that define priorities for future research. First, it examines a single, unusually well-documented pediatric oncology unit selected for theoretical leverage rather than representativeness. Comparative studies across specialties, hospitals, and health systems are needed to test whether the five boundary conditions proposed here travel beyond this case and whether different organizational arrangements produce different patterns of middle-up, middle-down, and sideways influence.

Second, the analysis relies primarily on implementation and operational records, which capture formalized rules, alerts, and recognized events more readily than informal coordination and off-platform work. Two authors’ involvement in developing and implementing AMIT provided unusually detailed access to rule provenance and implementation decisions, while also creating potential for interpretive bias; we therefore separated the coding role from the implementation role and made the coding rules explicit (Section 3.5 and Supplementary Material S3). Because the platform authors reviewed and approved the coding, one of whom adjudicated contested assignments; the coding relied on records produced by the platform team; and no coder outside the author team was used, the analysis is not fully independent of the implementation. Future studies combining digital traces with observation and interviews could capture both formal and informal middle-actor work.

Third, the study supports a mechanism rather than a causal-effect claim. The underlying evaluation was uncontrolled, so the clinical improvements reported by Singer et al. [24] may also reflect unmeasured confounders. Alert firing, override, false-positive, and false-negative rates were also not recorded, limiting the assessment of alert precision and possible VUCA amplification. The nursing survey was also small and single-site, had no pre-implementation baseline, and was distributed by the head nurse, which may have encouraged favorable responses despite anonymity; its findings should therefore be read as staff perceptions in this unit rather than as objective or generalizable measures of improvement. Future prospective studies should examine these measures alongside workload, rule maintenance, and professional responsibility.

Finally, planned large language model capabilities were not observed in practice. Their implementation offers a research setting for examining how middle actors mediate between algorithmically generated rules, clinical judgment, and institutional governance, and how this changes the distribution of agency and accountability.

## 6. Conclusions

This paper extends the VUCA framework and the Middle-Out Perspective into the high-acuity clinical environment of pediatric oncology, showing how nurses, ward managers and clinical leads, innovation staff, data architects, and the clinical safety team use a smart, rule-based platform (AMIT) to navigate volatility, uncertainty, complexity, and ambiguity in everyday practice. Re-coding the first 61 safety events the platform intercepted shows what such a platform manages: none arose from a gap in clinical knowledge, and all reflected failures in the availability, integrity, transfer, or timing of information the organization already held. This profile reflects what the platform is built to detect rather than all the risks in the unit. Digital decision-support tools can therefore be designed and governed as explicit middle-out VUCA-management instruments, strengthening frontline agency while introducing dependencies and risks. Read against published energy analysis, the case suggests that the framework travels under five clinical conditions, to be tested in other settings: compressed timescales; a bottom actor affected without participating; an instrument embedded in practice; delegated authority within a licensed hierarchy; and volatility met by redirecting attention within a fixed establishment rather than by adding capacity. In both settings, and as in public health, middle actors are not neutral [22]: enabling, mediating, and aggregating can stabilize or destabilize practice. Healthcare, particularly pediatric oncology, is a critical testbed for refining meso-level change theory and for design principles for smart platforms in VUCA-prone settings.

## Figures and Tables

**Table 1:** Key terms used.

Term	Meaning in This Paper
VUCA	Volatility, uncertainty, complexity, ambiguity: four distinct conditions that threaten performance, each calling for a different response [2] (Section 2.1).
Meso level	The organizational level of units, departments, teams, and professional groups, between the individual clinician–patient encounter (micro) and health-system policy (macro).
Middle actor	A group positioned between those who set the terms of care (top) and those who receive it (bottom), that controls a resource others need and has the will and means to use it [19,20]. Criteria in Section 3.4.
Middle-up, middle-down, sideways	The direction of a middle actor’s influence: toward management and institutional policy; toward frontline clinical work; toward peer professions and units.
Enabling, mediating, aggregating	The mode of influence: creating conditions or providing resources for others to act; negotiating or translating between actors or levels; integrating and institutionalizing information or initiatives [18,20].
Clinical decision support (CDS)	Software that delivers filtered, patient-specific information to clinicians at the point of decision [7].
Alert fatigue	Desensitization to alerts that are frequent and mostly irrelevant, leading clinicians to ignore or override them [8].
Poor transportability	Failure of rules or systems built at one site to work at another because systems, coding, and protocols differ [8].
E-iatrogenesis	Patient harm caused by the information technology itself, typically through implementation and workflow failures [7].
Boundary condition	A condition under which a theoretical framework holds, or must be modified, when transferred to a new setting.

**Table 2:** Representative clinical rules in AMIT.

Rule (Trigger)	Data Sources	Display	Expected Action
Neutropenia (absolute neutrophil count < 500/µL) with at least one abnormal vital sign	EHR and laboratory	Red margin around the patient’s row	Heightened vigilance and closer monitoring
Hemoglobin < 8 g/dL in a patient with leukemia	Laboratory and EHR	On-screen alert	Order red-cell transfusion
Hemoglobin < 9 g/dL in a patient with thalassemia	Laboratory and EHR	On-screen alert	Order red-cell transfusion
Invasive procedure scheduled by a physician: coagulation, platelet count, and anticoagulant prescriptions checked	Laboratory and EHR	On-screen alert	Hold the procedure until resolved
Patient receiving cisplatin: magnesium outside 2.0–2.4 mg/dL	Laboratory and EHR	On-screen alert	Correct magnesium or withhold the drug

Note. Alerts are displayed on the nursing-station dashboard to the unit’s nursing and medical staff. EHR, electronic health record.

**Table 3:** Sources of evidence and their relationship to the clinical evaluation by Singer et al. [24].

Source	Period Covered	Reported in Singer et al. [24]	Analysis Performed in This Study
Platform and rule documentation	2022–2025	Description of the platform	Coded by VUCA dimension, actor, direction, and mode (Figure 1); representative rules (Table 2)
Confirmed incident records	First 62 working days after full implementation (from October 2023)	61 events by clinical domain; two potentially life-threatening events	All 61 events re-coded by type of information failure (Section 4.2)
EHR operational data	Q4 2021–Q3 2025 (four phases)	Visits; urgent PICU referrals; time to antibiotics; interval from blood test to treatment; statistical tests	Reproduced as context only; not re-analyzed
Implementation and training documentation	January–September 2023	Implementation timeline	Coded as for platform documentation
Post-implementation nursing survey	July 2025	Summary proportions for selected items; qualitative comments	Full response distribution for all ten items (Section 4.8)

Note. EHR, electronic health record; PICU, pediatric intensive care unit; Q, quarter.

**Table 4:** The 61 intercepted safety events re-coded by type of information failure.

Type of Information Failure	Constituent Events	n	%
Latent result invisibility—a clinically significant result existed in a hospital system but was not present at the point and moment of decision	Invasive procedure with abnormal coagulation (7); procedure canceled for high bilirubin (4); abnormal renal function (1); abnormal liver enzymes (2); electrolyte disturbances requiring intravenous, oral, or community follow-up correction (11); high cortisol (1); high triglycerides (2); overlooked cytomegalovirus result (1)	29	47.5
Record integrity failure—the record did not correspond to the patient’s actual state	Height or weight typing errors (11); height or weight measurement errors (5); incorrect chemotherapy dose arising from an incorrect height (1); electrolyte result repeated and error ruled out (2); isolation events during the norovirus outbreak, where the record did not reflect the patient’s isolation status (9)	28	45.9
Coordination and hand-off failure—information held by another actor was not transferred	Anticoagulants taken by the patient and not known to the team (2)	2	3.3
Temporal validity failure—a recurring obligation was not surfaced when due, or an authorization remained in force after the information supporting it had changed	Monthly medication not administered on schedule (1); invasive procedure authorized two days in advance and not re-validated when critical data were missing (1)	2	3.3
Total		61	100

Note. Assignments follow the re-coding procedure in Section 3.5. Four groups of assignments, covering 14 events, were adjudicated against the underlying event records by the co-author who established and led the platform; Supplementary Material S3 sets out the reasoning for each. Decision rules: an event was assigned to latent result invisibility if a correct result existed in a hospital system but was absent at the point of decision; to record integrity failure if the recorded data did not match the patient’s actual state; to coordination and hand-off failure if the information was held by another actor and not transferred; and to temporal validity failure if a recurring obligation was not surfaced when due, or if an authorization outlived the information supporting it. The two potentially life-threatening events reported by Singer et al. [24], classified as such by a senior physician, fall in the first category: cisplatin, a nephrotoxic drug, was due to be administered to a patient with a glomerular filtration rate of about 30 mL/min/1.73 m^2^, and doxorubicin, which is cleared by the liver, to a patient with liver enzymes in the thousands. In both, administration risked irreversible renal or hepatic failure.

**Table 5:** Responses to the post-implementation nursing survey (n = 19).

Item (Abridged)	1	2	3	4	5	No Answer	4 or 5
1. How often do you use AMIT? ^a^	0	0	0	8	11	0	19/19
2. AMIT influenced my clinical day	0	0	2	7	10	0	17/19
3. AMIT improved workflow	0	0	2	7	10	0	17/19
4. AMIT improved my ability to prioritize clinical tasks	0	0	0	8	10	1	18/18
5. AMIT reduced the likelihood of missing critical medical data	0	0	0	9	10	0	19/19
6. AMIT increased my confidence in patient-management decisions	0	0	0	5	14	0	19/19
7. AMIT enhances clinical communication	0	0	2	6	11	0	17/19
8. AMIT decreases the end-of-shift bottleneck	0	0	2	8	9	0	17/19
9. AMIT enables faster patient transition	0	0	2	6	11	0	17/19
10. I would recommend AMIT to colleagues	0	0	0	5	14	0	19/19

Note. Number of respondents choosing each scale point (1 = strongly disagree, 5 = strongly agree). The last column gives the number of respondents choosing 4 or 5 out of those answering the item. Full item wording, in English translation, is given in Supplementary Material S2. ^a^ Frequency of use rather than agreement.

**Table 6:** Middle-out activities in the AMIT case that may amplify rather than reduce VUCA.

Middle Actor	Activity	Direction	Mode	Potential VUCA Risk	Explanation
ARC innovation staff/IT and data architects	Rapid addition of new alert rules as clinical requests accumulate	Middle-down	Enabling	Volatility, complexity	Rule proliferation without pruning increases screen noise, and acceptance falls as the alert stream thickens (Section 5.3). No firing or override data were recorded in this case, so proliferation could not be detected locally.
Ward managers and clinical leads/oncology nurses	Locally negotiated, ward-specific rule logic	Middle-down	Mediating	Complexity, ambiguity	Locally built rule logic solves the ward’s problem but can face interoperability and standardization barriers when carried to other systems, making otherwise sound platforms inefficient to disseminate and scale [8]; the same lack of standardization is reported across oncology decision-support tools [14].
ARC innovation staff	Presenting large before-after effect sizes to management to secure expansion	Middle-up	Aggregating	Uncertainty, ambiguity	Uncontrolled before-after improvements are not directly comparable with the pooled controlled-trial estimate of 5.8% [9]; over-claiming risks a hype cycle, misallocated investment, and disillusionment on scale-up—the clinical analogue of hype-driven cycles in energy innovation [25].
Clinical safety team	Confirming locally surfaced incidents as institutionally recognized safety events	Middle-up	Aggregating	Uncertainty	Only events the platform surfaces and staff report enter the count, so the recorded profile of informational failure reflects what AMIT makes visible rather than everything that occurs; the same aggregation that evidences the platform’s value also bounds it.
Oncology nurses	Sideways promotion of the platform to peer units (intended; Section 4.8)	Sideways	Mediating	Volatility, complexity	Diffusion ahead of maintenance capacity spreads a system whose rule base and data quality cannot be sustained locally. Maintenance is an often neglected part of the CDS life-cycle [8], and NASSS treats abandonment as an outcome of unmet adaptive capacity over time [13].
Ward managers and clinical leads	Delegating routine surveillance to the platform	Middle-down	Enabling	Uncertainty	Over-trust and deskilling: reliance on automation can erode independent scanning and verification skills, leaving staff less equipped when the system is unavailable or wrong [7,8].

## Data Availability

The data presented in this study are available on request from the corresponding author. The data analyzed here are internal implementation records and de-identified clinical safety records held by the participating medical center; they cannot be deposited in a public repository because they contain patient-level clinical information whose secondary use was approved for this analysis alone. The coding framework and the full coding protocol are provided in the Supplementary Material, and aggregated data underlying the reported figures are available from the corresponding author on reasonable request, subject to institutional approval. During the preparation and revision of this work the authors used ChatGPT (paid version; OpenAI, San Francisco, CA, USA) and Perplexity (Pro version; Perplexity AI, Inc., San Francisco, CA, USA) to assist with drafting, editing, and condensing the text. After using these tools, the authors reviewed and edited the content as needed and take full responsibility for the content of the publication. The use of these tools in the coding of the implementation records is described in Section 3.5 and Supplementary Material S3. Only de-identified material was processed with these tools. Perplexity was also used to prepare Figure 1 and the graphical abstract.

## Supplementary Materials

The following supporting information can be downloaded from: https://www.mdpi.com/article/10.3390/healthcare14213811/s1, Supplementary S1: Sources of evidence and access in detail; Supplementary S2: Post-implementation nursing survey instrument (Table S1: The ten items of the post-implementation nursing survey, English version of the Hebrew instrument.); Supplementary S3: Coding procedure in detail (Table S2: Codebook for VUCA dimensions, directions of influence, and modes of influence; Table S3: Codebook for the re-coding of the 61 intercepted safety events by type of information failure).
