# Supplementary material for: Dampening and Amplifying Clinical Uncertainty: Middle Actors and Rule-Based Decision Support in Pediatric Oncology
Source: Healthcare (Basel). 2026 Nov 8;14(21):3811. doi: 10.3390/healthcare14213811 (PMC13762623; doi:10.3390/healthcare14213811)
Supplement: Supplementary file 1 [file healthcare-14-03811-s001.zip › healthcare-4631743-supplementary.pdf]

Supplementary Materials

Dampening and Amplifying Clinical Uncertainty: Middle Actors and Rule-Based Decision Support in Pediatric Oncology

S1. Sources of evidence and access in detail

The four unit-record sources summarized in Section 3.2 are described here in full, together with the access on which the analysis rests. The fifth source, the post-implementation nursing survey, is summarized in Section 3.2 and reproduced in Supplementary Material S2.

(i) Platform and rule documentation: alert specifications, rationale, input systems, and rules added, modified, or withdrawn. (ii) Incident records: hospital-system reports of platform-surfaced events, reviewed and confirmed by the clinical safety team, with summary event descriptions and actions taken. (iii) Electronic health-record operational data: timestamped journey times—the interval between blood-test availability and corresponding medication or electrolyte administration—plus visits and referrals across four phases. (iv) Implementation and training documentation: clinical-team and innovation-center materials from development and training, including workflow descriptions. All available material of each type was considered; none was sampled. The incident records cover all confirmed events of the first 62 working days after full implementation in October 2023.

The author team's access to these materials provides individual-rule content and provenance, workflow adjustments, and responsibility distribution among nurses, physicians, and innovation staff—material normally invisible to external observers and absent from clinical evaluations. It also permits direct checking of middle-out readings against practice.

Two further details of the rule base described in Section 3.1 are documented. ARC, Sheba's innovation center, conducted a quality-assurance process directed at avoiding false negatives. One rule added after implementation directs AMIT to scan the prescriptions issued to a patient over the preceding three months.

S2. Post-implementation nursing survey instrument

The instrument reproduced below is the post-implementation nursing survey described in Section 3.2 and reported in Section 4.8. It was administered in Hebrew, anonymously and online through SurveyMonkey, to the twenty-two nurses of the unit, and was completed by nineteen of them, a response rate of 86.4%. The head nurse distributed the link by WhatsApp on 1 July 2025, and the survey remained open for three weeks. The items below are the English version of the instrument, titled "AMIT Satisfaction Survey". Each item was answered on a fully labeled five-point scale (1 = strongly disagree, 5 = strongly agree), which was explained in the written invitation and orally. The invitation read, in English translation: "To evaluate AMIT by those who use it, we have created a short survey of ten questions. Each question can be answered with a score from 1 to 5 (1 = strongly disagree, 5 = strongly agree); the other ratings are given in the table. The survey is anonymous and contains no identifying details, and answering it is not

mandatory. It serves us as a tool for obtaining feedback on AMIT. Thanks in advance to everyone who participates."

Table S1. The ten items of the post-implementation nursing survey, English version of the Hebrew instrument.

#	Question	Response scale (1 = strongly disagree, 5 = strongly agree)
1	How often do you use AMIT?	1 2 3 4 5
2	Do you feel that AMIT influenced your clinical day?	1 2 3 4 5
3	Did AMIT improve the workflow?	1 2 3 4 5
4	Did AMIT improve ability to prioritize clinical tasks?	1 2 3 4 5
5	Did AMIT reduce likelihood of missing critical medical data?	1 2 3 4 5
6	Did AMIT increase confidence in making patient management decisions?	1 2 3 4 5
7	AMIT enhances clinical communication	1 2 3 4 5
8	Does AMIT decrease bottleneck at the end of the shift?	1 2 3 4 5
9	Does AMIT enable faster patient transition?	1 2 3 4 5
10	Would you recommend your colleagues to implement AMIT?	1 2 3 4 5

Note. The full response distribution for all ten items is reported in Table 5 of the main text, where responses of 4 or 5 are treated as agreement. Item 1 records frequency of use rather than agreement.

S3. Coding procedure in detail

This section reproduces in full the coding procedure summarized in Section 3.5, and the adjudication of the contested event assignments recorded in the note to Table 4 of the main text.

Coding was carried out by the two authors who had no role in developing or implementing AMIT, with large language models (ChatGPT and Perplexity, paid versions) as assistants. All material processed with the models had been de-identified before it was made available for analysis, and no patient identifiers were entered into either tool. The procedure had three theory-guided steps. First, the model read the implementation records—rules, incidents, implementation and training documentation, and the nursing survey—and extracted excerpts relating to clinical challenges; AMIT design, logic, and alerts; workflow, role, and behavior changes; and reported outcomes and perceptions. The excerpts were compiled into a working dataset that retained both descriptions of context and evidence of responses. The operational data described in Section 3.2—visits, referrals, and journey times—were not coded; they are reported in the Results as context.

Second, the model assigned excerpts to the four VUCA dimensions using Bennett and Lemoine's (2014a, 2014b) distinctions [1,2] : rapid patient-status or workload changes as volatility; delayed or unseen results with known implications as uncertainty; fragmented information technology (IT) and overwhelming data as complexity; and emerging deterioration patterns or digital workflows with unclear "rules of the game" as ambiguity.

Third, the model coded VUCA-tagged excerpts for actors, influence directions (middle-up, middle-down, sideways), and modes (enabling, mediating, aggregating), following Janda and Parag (2013) [18], Parag and Janda (2014) [19], and Kranzler et al. (2019) [20]. The coded matrix underpins Figure 1 and the thematic narrative of the Results.

A further coding pass, reported in Section 4.2, addressed the 61 intercepted safety events. Working from the recorded event category, summary description, and action taken, and under the supervision of the same two authors, the model re-coded each event by the type of information failure underlying it rather than by clinical domain, producing the four categories reported in Table 4 of the main text. Four groups of assignments could not be settled from that material alone: the nine isolation events of the norovirus outbreak, the two events involving anticoagulants taken by the patient, the two repeated electrolyte results, and the invasive procedure authorized two days in advance. These were adjudicated by the co-author who established and led the platform, working from the underlying event records; the adjudication is set out at the end of this section. The two potentially life-threatening events had been classified as such by a senior physician in the original clinical evaluation.

Consistent with the procedure in Parag and Yogev (2026) [25], iterative prompt refinement with human-in-the-loop supervision, not model training, was used. The authors validated the output at each step: proposed excerpts and category assignments were checked against the original material and were accepted, revised, or rejected. Final coding decisions are the authors' and rest on theory and clinical expertise. The two authors who developed AMIT then reviewed and approved the coded assignments and findings. No coder outside the author team was used, so no inter-rater agreement statistic is available, as noted in Section 5.6 of the main text.

Adjudication of the contested assignments in Table 4. Four groups of assignments, covering 14 events, were adjudicated as follows. The nine isolation events of the norovirus outbreak are classified as record integrity failures, because the record did not reflect the patients' isolation status; the two anticoagulant events as coordination and hand-off failures, because the medication was known to the patient but was neither present in the electronic record nor elicited by the team; the two repeated electrolyte results as record integrity failures; and the invasive procedure authorized two days before it was performed as a temporal validity failure, since the authorization remained in force after the data supporting it had ceased to be current. This last event fitted none of the original four categories, and the fourth category was widened accordingly.

Codebook. Tables S2 and S3 give the decision rule and an example from the case for each category used in the coding.

Table S2. Codebook for VUCA dimensions, directions of influence, and modes of influence.

Category	Decision rule	Example from the case
**VUCA dimension**		
Volatility	Rapid change, whose causes are understood, in patient status, caseload, or acuity that requires reprioritization	Clinical deterioration of a neutropenic child during a day care visit
Uncertainty	Known implications but unclear timing or visibility of information: whether, when, and to whom a result becomes known	A culture or viral-load result returning days after the visit, when the child is at home
Complexity	Many interconnected systems, information sources, or workflow steps that overload decision makers	Checking seven unintegrated hospital systems before administering chemotherapy
Ambiguity	Unclear cause–effect relationships or no precedent: which signals indicate decline, and how alerts fit existing responsibilities	Deciding which combination of vital signs and laboratory values should trigger a red margin
**Direction**		
Middle-down	Influence on frontline clinical work, individual decisions, or episodes of care	Rules that reorder nurses' task lists
Middle-up	Influence directed at hospital management or institutional policy	Journey-time evidence presented to management to sustain the platform
Sideways	Influence directed at peer professions or units at the same level	Nurses escalating alerts to physicians and the PICU
**Mode**		
Enabling	Creating conditions, or providing tools or resources, that allow others to act	Real-time alerts and red margins for high-risk patients
Mediating	Negotiating, brokering, or translating between actors or levels	Co-designing ward-specific rules with ward managers
Aggregating	Gathering, integrating, and institutionalizing information or initiatives	Compiling late-arriving results into a prioritized follow-up list

Note. PICU, pediatric intensive care unit.

Table S3. Codebook for the re-coding of the 61 intercepted safety events by type of information failure.

Category	Decision rule	Example from the case
Latent result invisibility	A clinically significant result existed in a hospital system but was absent at the point and moment of decision	Chemotherapy due to be given despite an abnormal renal-function result
Record integrity failure	The recorded data did not correspond to the patient's actual state	Isolation status during the norovirus outbreak not reflected in the record
Coordination and hand-off failure	Information held by another actor, including the patient, was not transferred to those who needed it	An anticoagulant taken by the patient but absent from the record and not elicited
Temporal validity failure	A recurring obligation was not surfaced when due, or an authorization remained in force after the information supporting it had changed	An invasive procedure authorized two days in advance on data no longer current

Note. Each event received a single category.
